# High-frequency ultrasound characterization of vulvar skin in patients with lichenoid vulvar dermatoses and correlation of its vascular index with microvessel density and microvessel area

**DOI:** 10.3389/fmed.2025.1661619

**Published:** 2026-01-07

**Authors:** Jiyun Chen, Kaikai Shen, Jun Xv, Yujie Shi, Qiuyu Liu, Haohui Zhu, Xijun Zhang

**Affiliations:** 1 Department of Ultrasound, Henan Provincial People’s Hospital, Zhengzhou, China; 2 Department of Obstetrics and Gynecology, Henan Provincial People’s Hospital, Zhengzhou, China; 3 Department of Pathology, Henan Provincial People’s Hospital, Zhengzhou, China

**Keywords:** high-frequency ultrasound, lichenoid vulvar dermatoses, microvessel area, microvessel density, vascular index

## Abstract

**Objectives:**

We utilized high-frequency ultrasound to characterize vulvar skin alterations in lichenoid vulvar dermatoses (LVD) and to determine whether the sonographic vascular index (VI) correlates with histopathological microvessel density (MVD) and microvessel area (MVA).

**Methods:**

This study included thirty-seven patients with pathologically confirmed LVD who attended Henan Provincial People’s Hospital between December 2021 and May 2024. A control group of thirty-five healthy women, matched for age and BMI was selected during the same period. High-frequency ultrasound parameters of the vulvar skin between the two groups were analyzed using statistical tests. Vulvar skin specimens from LVD patients were stained with CD34 to determine MVD and MVA, and the correlation with the VI from ultrasound parameters was assessed.

**Results:**

(i) Analysis of variance showed that epidermal thickness, subepidermal low echogenic band (SLEB) thickness, dermal thickness, and VI were significantly increased in the LVD group compared to the control group (all *P* < 0.05). (ii) Correlation analysis revealed a positive correlation between the VI and MVD, MVA (*r* = 0.438, *r* = 0.630, both *P* < 0.05).

**Conclusion:**

The vulvar skin thickness and VI in patients with LVD are increased compared to the control group. The VI reflects the microvascular status in LVD, highlighting the significant value of high-frequency ultrasound in assessing LVD.

## 1 Introduction

Lichenoid vulvar dermatoses (LVD) are a group of chronic, recurrent, inflammatory skin conditions primarily characterized by itching. They predominantly affect the genital and anal regions, presenting clinical features such as hypopigmentation, skin and mucous membrane thickening, and atrophy (1, 2). If left untreated, LVD can lead to a loss of normal vulvar architecture or even progression to vulvar squamous cell carcinoma, severely impacting the patient’s quality of life (3). Therefore, early diagnosis, prompt treatment, and continuous disease monitoring during therapy are essential.

Pathologic biopsies are commonly used to diagnose and assess skin changes in LVD patients. However, their clinical application is limited by their invasive nature. High-frequency ultrasound (HFUS), known for its real-time, non-invasive imaging capabilities, is widely used for evaluating various cutaneous diseases (4). Additionally, superb microvascular imaging (SMI), an advanced Doppler technique, capable of visualizing enhances this by providing clear visualization of low-velocity microvessels (5). The vascular index (VI) offers a quantitative and convenient method for measuring the proportion of microvascular areas, providing valuable insights into the activity levels of inflammatory diseases (6).

Angiogenesis is a hallmark of chronic inflammation, with both processes being interdependent (7). CD34, a marker expressed by both mature and newly formed vascular endothelial cells (8), is commonly used to evaluate microvascular changes. Microvessel density (MVD) and microvessel area (MVA) are well-established parameters for assessing angiogenesis levels.

In this study, we employed HFUS to evaluate vulvar changes in patients with LVD. Furthermore, we investigated the correlation between the VI obtained from HFUS and the histopathological parameters of MVD and MVA. These analyses were conducted to assess the feasibility and accuracy of using HFUS and the VI as diagnostic tools.

## 2 Materials and methods

### 2.1 Study population

Thirty-seven patients with LVD, attending the outpatient clinic of the Department of Obstetrics and Gynecology at Henan Provincial People’s Hospital from December 2021 to May 2024, were selected as the LVD group. The inclusion criteria were: (1) confirmed LVD diagnosis via pathological biopsy; (2) aged 18 or older; (3) voluntary consent for ultrasonic examination. The exclusion criteria were: (1) treatment with physicotherapeutics, glucocorticoids or tacrolimus within the past 3 months; (2) the presence of other vulvar diseases, such as psoriasis; (3) pregnancy or lactation; (4) refusal to undergo pathological biopsies.

Thirty-five healthy adult females, matched for BMI and age, attending the Health Management Discipline at Henan Provincial People’s Hospital during the same period, were selected as the control group.

Vulvar biopsies of LVD patients were collected and stained with CD34. However, due to the invasive nature of pathological biopsies, control group participants only underwent ultrasound examinations without biopsy. The study protocol was approved by the ethics committee of Henan Provincial People’s Hospital, and informed consent was obtained from all participants.

### 2.2 Ultrasound examination

**FIGURE 1:**
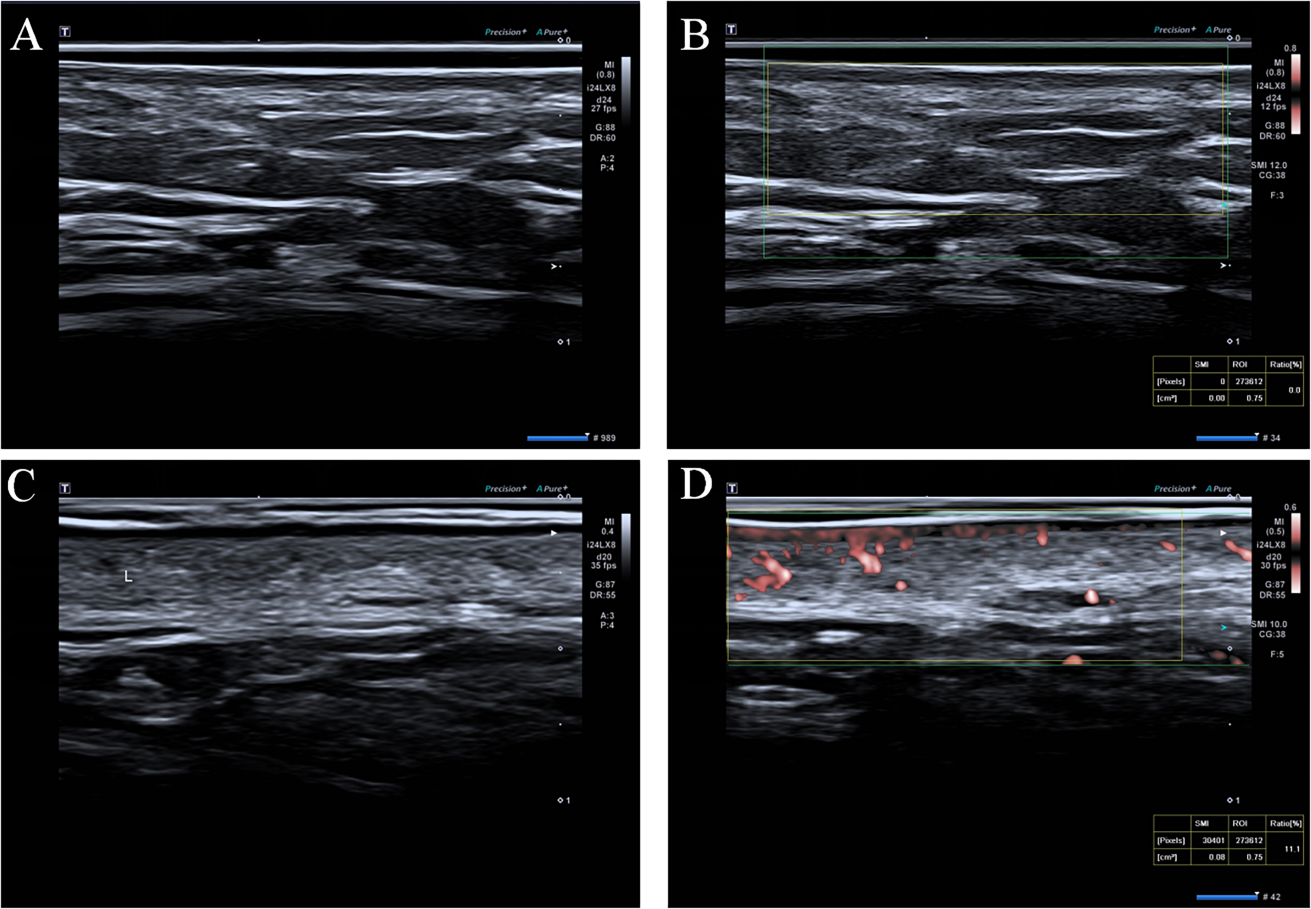
**(A)** Gray-scale ultrasound images of the control group; **(B)** VI measurement image of microvascular blood flow for the control group; **(C)** Gray-scale ultrasound images of the LVD group; **(D)** VI measurement image of microvascular blood flow for the LVD group.

The Canon Aplio i900 ultrasonic diagnostic instrument (Canon Medical Systems Corporation, Japan) and PLI-2004BX linear probe (probe frequency 8∼24 MHz) were used for skin imaging. The SKIN mode was selected and the gain was adjusted to clearly visualize the epidermis, dermis, and subcutaneous tissue. Patients were positioned in the lithotomy position to fully expose the perineal area. The probe was enveloped in a disposable film and an appropriate amount of ultrasound gel was applied. The examination was conducted with the probe held perpendicular to the perineal area. During the examination, the vulva was comprehensively scanned using an ultrasound probe, ranging from the mons pubis to the posterior commissure. Select regions with abundant blood flow for imaging acquisition. Mark these areas post-ultrasound to ensure consistency between imaging and tissue sampling sites. Images were captured and stored when they are stable and clear (Figure 1). Using ImageJ 2.0 software (National Institutes of Health, USA), measurements were taken of the epidermal thickness, subepidermal low echogenic band (SLEB) thickness, and dermal thickness. SMI mode was activated with the color gain set to a fixed value of 38 dB, to observe the blood vessels within the dermis. The image showing the richest and most abundant blood flow signal was selected, and a region of interest (ROI) was drawn to obtain the VI.

Ultrasound parameters were measured three times, with the mean value recorded as the final result.

### 2.3 CD34 staining and measurement of MVD and MVA

**FIGURE 2:**
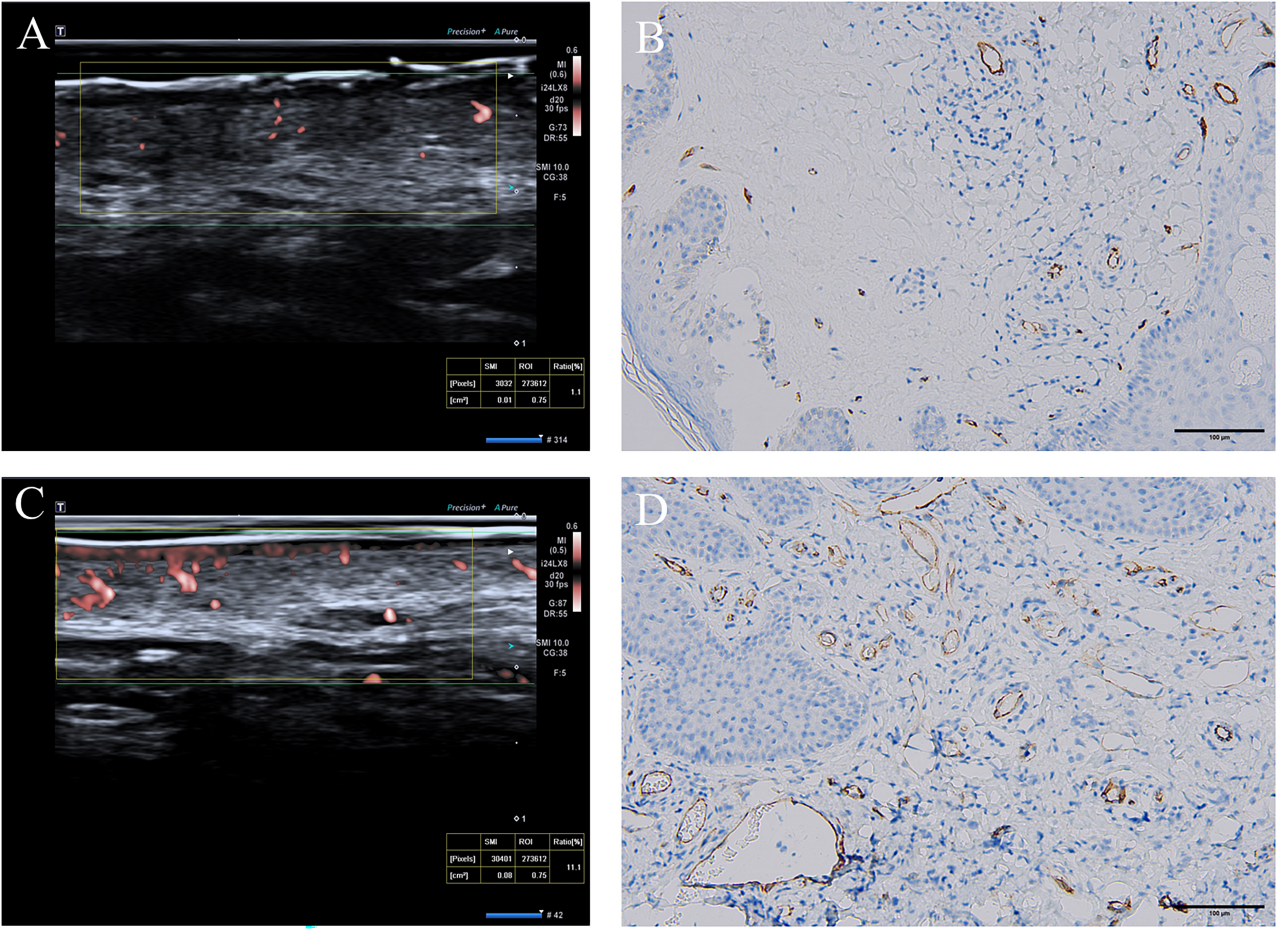
**(A)** VI measurement image of microvascular blood flow for the LVD group; **(B)** CD34 staining in the same LVD patient as in panel **(A)**, ×200 magnification; **(C)** VI measurement image of microvascular blood flow for the LVD group; **(D)** CD34 staining in the same LVD patient as in panel **(C)**, ×200 magnification.

Pathologic biopsy and CD34 staining were conducted at the same site of the HFUS examination. Prior to biopsy, local subcutaneous infiltration anesthesia was administered using 1% lidocaine. A surgical blade was used to make a spindle-shaped incision into the skin, with a depth of approximately 1 cm, to obtain a specimen comprising epidermis, dermis, and partial subcutaneous tissue. Hemostasis was achieved by compression, followed by a single absorbable suture if necessary. The acquired tissue samples were promptly sent to the pathology department, where pathological diagnosis and CD34 immunohistochemical staining were performed by a pathologist. All CD34-stained slides were examined using an Olympus microscope (BX53, Olympus Corporation, Japan). Blood vessels were identified as CD34-stained, brownish-yellow lumens without a muscular wall. MVD and MVA were assessed using the Weidner (9) method. Initially, the entire area was studied at ×40 magnification to identify “hot spots.” Then, at ×200 magnification, three images with a high blood vessel count were captured, and their average was calculated to determine MVD (Figure 2). These high-magnification images were then imported into ImageJ (National Institutes of Health, USA) software for manual tracing of microvascular regions, which were summed to calculate the MVA for each image. The three highest MVA values for each patient were averaged to obtain the final MVA.

### 2.4 Statistics analysis

Data analysis was conducted using software SPSS version 27.0 (IBM, Armonk, NY, USA). Continuous data conforming to normal distribution were expressed as mean ± standard deviation, with group differences assessed using independent samples *t*-test (for homogeneity of variance) and *t*’-test (for inhomogeneity of variance). Continuous data not conforming to normal distribution were expressed as median (interquartile range), with group differences evaluated using the Mann-Whitney U test. Correlations between VI parameters and MVD, MVA were determined using Pearson’s or Spearman’s correlation coefficients. A two-tailed *P*-value < 0.05 was considered statistically significant.

## 3 Results

### 3.1 Clinical parameters

Thirty-seven patients with LVD were included in this study, all of whom were female. The control group consisted of thirty-five adult females. The age range of patients in the LVD group was 20–66 years, with a mean age of 38.62 ± 10.07 years. Healthy individuals in the control group had an age range of 20–63 years and a mean age of 37.83 ± 11.91 years. There was no statistically significant age difference between the two groups (*t* = 0.306, *P* = 0.761). The disease duration in the LVD group ranged from 6 to 130 months, with a median of 29 months. In the LVD group, there were 26 pre-menopausal patients (70.27%), 8 peri-menopausal patients (21.62%), and 3 pos-tmenopausal patients (8.11%). In the control group, there were 26 pre-menopausal patients (74.28%), 4 peri-menopausal patients (11.43%), and 5 post-menopausal patients (14.29%). The chi-square test revealed no statistically significant difference between the two groups (χ^2^ = 1.809, *P* = 0.405).

### 3.2 Ultrasound parameters

The thickness of epidermis, SLEB and dermis in the LVD group was significantly increased than that in the control group (all *P* < 0.05). Additionally, the VI was significantly higher in the LVD group (*P* < 0.05) compared to the control group. The specific parameters were presented in Table 1.

**TABLE 1:** Ultrasound parameters.

Ultrasound parameters	LVD group (*n* = 37)	Control group (*n* = 35)	*t’/Z*	*P*-value
Epidermal thickness (mm)	0.25 ± 0.06	0.20 ± 0.04	4.607	<0.001
SLEB thickness (mm)	0.18 (0.16)	0.00 (0.06)	−5.552	<0.001
Dermal thickness (mm)	1.38 ± 0.46	0.82 ± 0.17	6.877	<0.001
VI	4.23 (5.59)	0.77 (0.37)	−5.354	<0.001

LVD, lichenoid vulvar dermatoses; SLEB, subepidermal low echogenic band; VI, vascular index.

### 3.3 Correlation between VI and MVD, MVA

**FIGURE 3:**
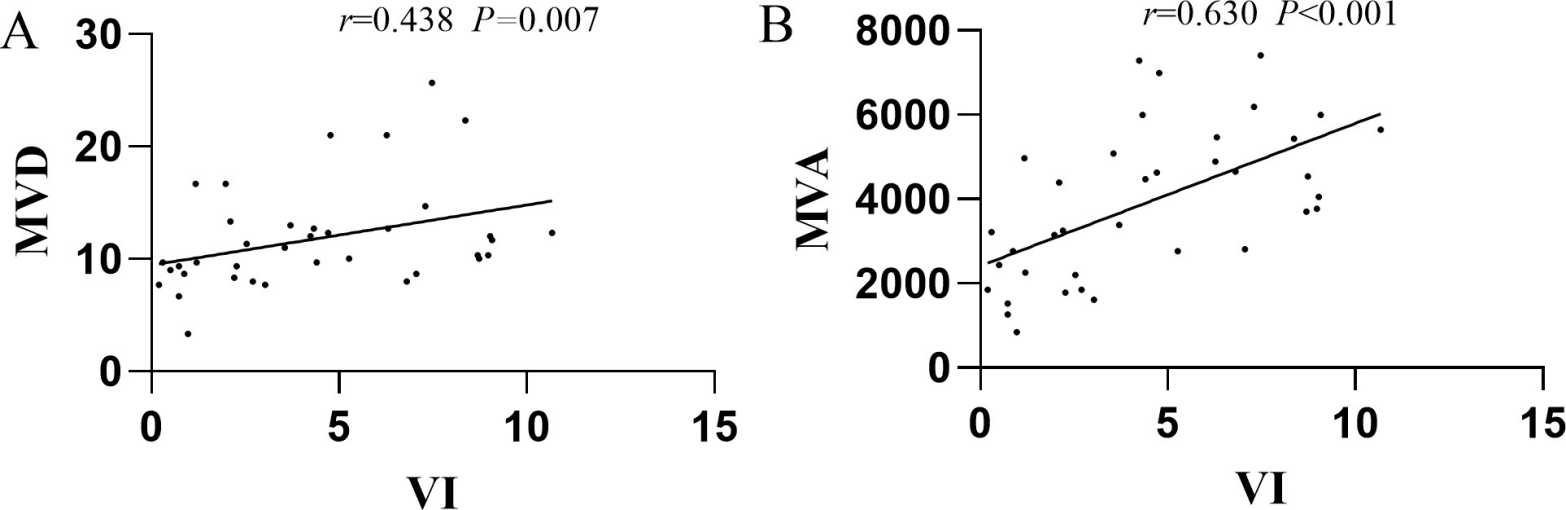
**(A)** Correlation of MVD with VI; **(B)** correlation of MVA with VI. MVD, microvessel density; MVA, microvessel area; VI, vascular index.

Spearman correlation analysis revealed a weak correlation between VI and MVD (*r* = 0.438, *P* = 0.007). A moderate correlation was observed between VI and MVA (*r* = 0.630, *P* < 0.001), as shown in Figure 3.

### 3.4 Repeatability test

Ultrasound images and high magnification images stained by CD34 from fifteen patients were randomly selected for reproducibility testing. Two observers, including one repeated observer, measured the ultrasound images with a 1-week interval between measurements. The intraclass correlation coefficients (ICC) were used to evaluate the intra- and inter-observer agreement for each parameter, with good consistency shown in Table 2 (ICC > 0.75, *P* < 0.05).

**TABLE 2:** Repeatability test.

	Intra-observer reliability		Inter-observer reliability	
Parameter	ICC	95% CI	ICC	95% CI
Epidermis	0.949	0.821–0.987	0.939	0.753–0.985
SLEB	0.966	0.871–0.991	0.945	0.805–0.986
Dermis	0.976	0.912–0.994	0.969	0.879–0.992
MVD	0.972	0.896–0.993	0.968	0.878–0.992
MVA	0.983	0.935–0.996	0.972	0.898–0.993

ICC, intraclass correlation coefficients; CI, confidence intervals; SLEB, subepidermal low echogenic band; MVD, microvessel density; MVA, microvessel area.

## 4 Discussion

High-frequency ultrasound is extensively utilized for detecting various skin diseases, due to its ability to visualize the structures of different skin layers (10). In this study, we utilized HFUS to assess vulvar changes in women with LVD. Our findings revealed that, compared to the control group, patients with LVD exhibited increased vulvar skin thickness and a higher VI. Furthermore, within the LVD patient group, a positive correlation was observed between the VI and both MVD and MVA.

The thickness of healthy adult skin is influenced by various factors, including age, gender, and photoaging (11). In this study, all subjects were female with vulvar tissues, and there was no statistically significant age difference between the control and LVD groups. Therefore, we attribute the observed difference in skin thickness to the disease itself. LVD encompasses a group of inflammatory skin diseases, including vulvar lichen sclerosus (VLS), vulvar lichen simplex chronicus (VLSC) and vulvar lichen planus (VLP) (1). Cases of VLS (19 cases) and VLSC (18 cases) were included in the LVD group. However, due to the limited number of CD34-stained vulvar tissues, a detailed subgroup analysis was not performed.

Histopathologically, VLSC is characterized by epidermal thickening, hyperkeratosis, spongiosis, acanthosis, and a dermal infiltrate of inflammatory cells (12, 13). The features of VLS include a thickened or diminished granular layer, atrophic epidermis, diffuse dermal sclerosis, and an interstitial pattern of inflammation (14). Current research on epidermal changes in LVD remains limited and inconsistent. A study (15) found that, compared to the control group, the full epidermal and cell-layer thickness did not significantly change in early VLS, but the cell-layer thickness decreased in late VLS. Our research findings, however, demonstrated increased epidermal thickness in LVD, which we speculate may be attributed to the epidermal thickness of VLSC and the number of cases, offsetting the differences in epidermal thickness observed in VLS.

The SLEB is a distinctly visible hypoechoic band located between the lower epidermal layer and the upper dermal layer. Its thickness is affected by factors like age and photoaging and is associated with the dermal infiltration of inflammatory cells (16). Our study revealed a statistically significant difference in SLEB thickness in the vulvar lesion area between patients with LVD and the control group, aligning with previous research (17, 18). Inflammatory diseases such as psoriasis, rosacea, and lichen planus, also exhibit thickening of the SLEB (15), which is attributed to inflammatory cell infiltration in the dermis. This infiltration damages the dermal-epidermal junction and results in SLEB thickening (19, 20). Moreover, dermal inflammation can cause edema and as the disease progresses, deeper inflammatory cell infiltration and dermal hardening occur, further increasing dermal thickness (17). In summary, the increased SLEB thickness and dermal thickness observed in our LVD patients are consistent with the classic ultrasonic characteristics of lichen planus as previously described in the literature (21).

Lichenoid vulvar dermatoses is a chronic, inflammatory skin disease with an unknown pathogenesis, in which angiogenesis plays a key role. Inflammation stimulates angiogenesis, which in turn exacerbates chronic inflammation, creating a self-perpetuating cycle.

In summary, neovascularization is a hallmark of chronic inflammation (7, 8). We analyzed MVD and MVA to quantify angiogenesis in the vulvar skin of patients with LVD. Concurrently, we employed a novel high-frequency ultrasound technology known as SMI, which uses the VI to assess disease activity (6) by measuring the proportion of the vascular area.

Our study revealed that the VI was higher in the LVD group than in the control group, consistent with previous research (22, 23). Furthermore, VI showed a moderate correlation with MVA (*r* = 0.630), but only a weak correlation with MVD (*r* = 0.438). The absence of correlation between VI and MVD is attributed to their different measurement principles: VI, being a ratio (24), essentially represents an area, whereas MVD considers only the count, disregarding the area. Regardless of whether MVA or MVD is considered, our findings suggest that VI is a feasible tool for assessing blood flow in LVD and holds value for disease assessment and monitoring.

This study has several limitations. Firstly, the study is limited by its small sample size and cross-sectional design. Secondly, the analysis was not stratified by key variables such as age and disease duration, which prevents an assessment of how these factors influence the ultrasound parameters. Thirdly, while two types of LVD were included, a detailed comparison between them was not conducted. Future studies could include a larger sample size and more types of LVD to further investigate their high-frequency ultrasound features and the correlation between ultrasound-assessed epidermal and dermal thicknesses with pathological measurements, thereby enhancing the applicability of high-frequency ultrasound.

This study utilized high-frequency ultrasound to assess the changes in the vulvar skin of patients with LVD, and to examine the correlation between the vascular index observed under ultrasound and the microvasculature identified through pathology. The findings established the high-frequency ultrasound characteristics and demonstrated the accuracy of blood flow parameters for LVD patients, providing a new non-invasive method for the assessment and follow-up of LVD in the future.

## Data Availability

The raw data supporting the conclusions of this article will be made available by the authors, without undue reservation.
